# Detecting Pathogenic *Phytophthora* Species Using Volatile Organic Compounds

**DOI:** 10.3390/molecules29081749

**Published:** 2024-04-12

**Authors:** Patrick Sherwood, Ida Nordström, Steve Woodward, Björn Bohman, Michelle Cleary

**Affiliations:** 1 Southern Swedish Forest Research Centre, Swedish University of Agricultural Sciences, Box 190, 234 22 Lomma, Sweden; ida.nordstrom@slu.se (I.N.); michelle.cleary@slu.se (M.C.); 2 Department of Plant and Soil Science, School of Biological Sciences, University of Aberdeen, Cruickshank Building, St. Machar Drive, Aberdeen AB24 3UU, UK; s.woodward@abdn.ac.uk; 3 Department of Plant Protection Biology, Swedish University of Agricultural Sciences, Box 190, 234 22 Lomma, Sweden; bjorn.bohman@slu.se

**Keywords:** gas chromatography–mass spectrometry, tree disease, volatilomics, *Fagus sylvatica*, *Quercus robur*

## Abstract

There are several highly damaging *Phytophthora* species pathogenic to forest trees, many of which have been spread beyond their native range by the international trade of live plants and infested materials. Such introductions can be reduced through the development of better tools capable of the early, rapid, and high-throughput detection of contaminated plants. This study utilized a volatilomics approach (solid-phase microextraction coupled to gas chromatography–mass spectrometry) to differentiate between several *Phytophthora* species in culture and discriminate between healthy and *Phytophthora*-inoculated European beech and pedunculate oak trees. We tentatively identified 14 compounds that could differentiate eight *Phytophthora* species from each other in vitro. All of the *Phytophthora* species examined, except *Phytophthora cambivora*, uniquely produced at least one compound not observed in the other species; however, most detected compounds were shared between multiple species. *Phytophthora polonica* had the most unique compounds and was the least similar of all the species examined. The inoculated seedlings had qualitatively different volatile profiles and could be distinguished from the healthy controls by the presence of isokaurene, anisole, and a mix of three unknown compounds. This study supports the notion that volatiles are suitable for screening plant material, detecting tree pathogens, and differentiating between healthy and diseased material.

## 1. Introduction

*Phytophthora* is an extremely important genus of plant pathogens responsible for massive economic losses and ecological damage in agriculture, horticulture, and forestry [1,2]. Currently, approximately 200 species of *Phytophthora* are known, but it has been estimated that the total number of species globally is likely to be 600 or more [3]. The host ranges of *Phytophthora* spp. vary greatly, but as an example, *Phytophthora cinnamomi* has a host range close to 5000 species of plants, including many of importance in agriculture, forestry, and horticulture [4,5]. As tree pathogens, *Phytophthora* are most damaging as root rots and stem cankers, but they can also cause foliar blights. Infection can lead to reduced growth, plus an increased sensitivity to drought, herbivores, and other stresses [4,5,6], the decline and death of individual trees, and even widespread mortality in the landscape [1]. Their diverse host ranges, persistence in soil once introduced, and potential for highly damaging outbreaks have made *Phytophthora* species some of the most important plant pathogens regarding plant health and management practices.

Many of the most problematic *Phytophthora* spp. are so because they are invasive alien species (IASs) and the native flora lack sufficient and evolved defenses against them [7]. Economic and ecological losses stemming from the introduction of IASs have been recognized as an increasingly difficult challenge worldwide in agriculture, horticulture, and forestry [8,9]. The spread of plant pathogens that can potentially become invasive is enabled foremost by the international plant trade, which has increased substantially in recent decades [10], with no signs of slowing down [11]. *Phytophthora* spp. are common IASs found in the plant trade [12], often in the soil and compost used in the production of hardy woody nursery stock [13], making them difficult to detect by traditional methods in shipments of asymptomatic potted plants. Moreover, in nurseries, large numbers of plants are raised in restricted spaces, promoting the survival and proliferation of *Phytophthora* spp. due to the high host plant availability, regular irrigation, and favorable temperatures, with the consequence of the pathogens accompanying the plants to their final planting positions. Numerous introductions of *Phytophthora* spp. into forest environments have occurred over many years in this way [12]. This problem can be reduced by the implementation of suitable management and prevention practices and novel detection techniques in trade and at ports of entries [14,15]. Proactive strategies and the development of advanced pathogen detection methods could greatly improve our capacity to mitigate the infiltration of invasive alien pathogens in international and national trade and limit their introduction to new areas.

Nucleic-acid-based techniques for detecting plant pathogens are improving rapidly [16]. For example, loop-mediated isothermal amplification and nanopore sequencing can be used on-site for point-of-need detection [17,18], while the long-read sequencing capabilities of PacBio and Oxford Nanopore Technologies grant a better taxonomic resolution for regions like the fungal internal transcribed spacer [19]. Despite these improvements, molecular methods still have limited utility in plant biosecurity due to (i) the extensive volume of plants traded internationally, which overwhelms the staffing levels at entry ports, limiting the ability to conduct comprehensive inspections on a significant portion of the units in transit [15,20], (ii) limitations in detection, in that DNA analyses require the destructive sampling of the correct tissues on the plants or the infested compost, and (iii) the need for molecular and bioinformatics proficiencies in ensuring the correct sample processing. Hence, novel non-DNA-based approaches better tailored for early and high-throughput detection are needed. Such approaches could also serve as initial screening tools when combined with more targeted molecular techniques.

Plants release a multitude of volatile organic compounds (VOCs) into their immediate environment, which fulfill critical roles in growth, intra- and interspecific communication, defense, and survival [21]. The composition of emitted VOCs, akin to distinct chemical fingerprints, dynamically varies among plant species and may differ in each plant–pathogen interaction, offering a potential utility for VOCs as indicators of plant health [21,22]. For example, VOCs produced by plant pathogens are already targeted for the detection of foodstuff spoilage in agriculture [22,23]. In forestry, VOCs-based detection methods are less researched and have not yet been implemented commercially. Recently, Nordström et al. [24] successfully distinguished *Fusarium circinatum*-infected *Pinus* spp. seedlings from healthy ones, and this study also revealed that each included *Fusarium* spp. showed discernible VOC blends, even when cultivated on the same substrate. Vuorinen et al. [25] pointed to the potential of VOCs as pathogen-specific disease indicators, as *Betula* spp. trees exposed to pathogens could be distinguished from those affected by herbivores. In addition, Johne et al. [26] could distinguish between pathogenic fungi in *Aesculus* spp. in infected oak acorns, and Borowik et al. [27] were able to distinguish between *Phytophthora plurivora* and *Pythium intermedium* using a VOCs-based detection method.

This project was devised as two separate but complementary experiments. The first experiment examined the in vitro VOCs of multiple *Phytophthora* spp., with the aim of determining whether an analysis of VOCs can be used to distinguish species and generate potential VOC biomarkers for the tested species. The second experiment examined the in vivo VOCs of stem-inoculated trees to ascertain whether infected trees could be distinguished from healthy controls. For this work, we used solid-phase microextraction (SPME) fibers in conjunction with gas chromatography–mass spectrometry (GC-MS) to examine the VOCs from eight *Phytophthora* species when grown in vitro, and differences in *P. cinnamomi*- and *P. plurivora*-infected pedunculate oak (*Quercus robur*) and European beech (*Fagus sylvatica*) were compared to mock-inoculated controls (MIC). Volatilomics using SPME is a versatile technique commonly used for the static capture of VOCs in biological systems, because it is an economical, simple, and non-destructive sampling strategy that can capture a large fraction of the full volatilome [28]. Combining SPME and GC-MS is, therefore, useful and germane in a screening study like this, where the objective is to discover biomarkers of disease that can be used in targeted methods for disease detection and diagnosis in the future.

## 2. Results

### 2.1. In Vitro Study

The objective of the in vitro study was to discover *Phytophthora*-related volatiles that were qualitatively different from the media controls. In total, we found 58 compounds from the *Phytophthora* species (isolates listed in Table S1) that were not in the media-only control vials; a list of these compounds is presented in Table 1. There was a similar number of *Phytophthora* compounds detected at both collection time points, 14 days post-inoculation (dpi) and 30 dpi. In total, 43 compounds were detected at 14 dpi and 46 compounds at 30 dpi. Of the 58 total compounds, 31 were observed at both time points, while 12 were only detected at 14 dpi and 15 were only detected at 30 dpi. There was considerable variability in the number of compounds observed between the *Phytophthora* species examined (Table 1). *Phytophthora gonapodyides* and *P. polonica* had the most compounds detected with 25 each, *P. cambivora* was next with 22, followed by *P. multivora* with 18. Meanwhile, nine compounds were detected from *P. plurivora*, eight from *P. cinnamomi*, five from *P. citricola*, and only three from *P. syringae*. *Phytophthora plurivora* had all nine of its detected compounds occurring at both the 14 and 30 dpi time points. *Phytophthora cinnamomi* had all but one of its eight compounds occurring at both time points. *Phytophthora cambivora*, *P. gonapodyides*, and *P. polonica* had the most differences in the number of compounds between time points. In *Phytophthora cambivora* and *P. gonapodyides*, 13 and 17 compounds, respectively, were uniquely present at the 30 dpi time point, while *P. polonica* had 14 compounds present only at the 14 dpi time point.

**Figure 1:**
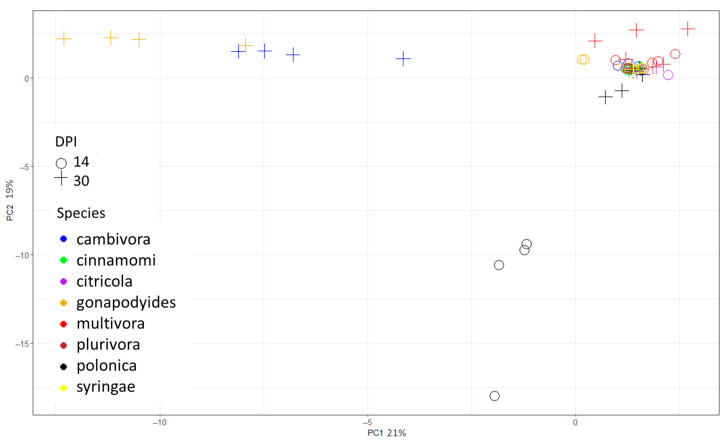
Principal component analysis (PCA) components plot of volatile compounds detected at 14 and 30 days post-inoculation (DPI) from eight *Phytophthora* species grown in vitro. N = 4 for each species by time point.

All *Phytophthora* species, except *P. cambivora*, had at least one exclusive VOC. *Phytophthora polonica* had the highest number of compounds only found in a single species, with 12 compounds, *P. multivora* had 7, *P. gonapodyides* had 6, and *P. cinnamomi* had 2, while *P. citricola*, *P. plurivora*, and *P. syringae* each had 1. Since most of the examined species had only a few unique compounds (many of which were specific to a certain time point), multivariate analyses were run on the full compound list in Table 1. The PCA demonstrated that some *Phytophthora* species can be distinguished based on VOCs (Figure 1). At 30 dpi, *P. cambivora* and *P. gonapodyides* were well separated from the other species via PC1, but did not separate well from each other. *Phytophthora polonica* was separated from the other species at the 14 dpi time point, predominantly via PC2. The remaining species and time points had a poor resolution, with only *P. multivora* at 30 dpi showing some separation. The top five loadings for PC1 and PC2 are listed in Table 2.

**Figure 2:**
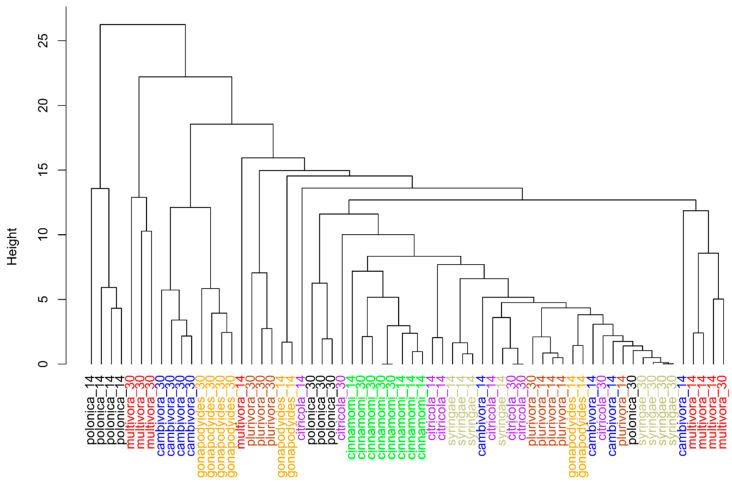
Scaled hierarchical cluster analysis of *Phytophthora* species based on the volatile compounds detected at 14 and 30 days post-inoculation. Species names are abbreviated to the specific epithet and are followed by the numeral for the sampling date post inoculation.

A cluster analysis largely corroborated the PCA, as *P. polonica* (at 14 dpi), *P. gonapodyides*, *P. cambivora*, and *P. multivora* (all at 30 dpi) tended to form distinct clusters with greater separation from the other species based on node height (Figure 2). The other species had shorter branch lengths and lower node branching points, indicating that they were more similar. For all species, except *P. citricola*, replicates did generally cluster by species and sampling time.

The five most important compounds for discriminating between *Phytophthora* species, according to the random forest analysis based on mean Gini scores, are listed in Table 2 (see Table S2 for the full random forest analysis results and Figure S1 for mass spectra for unknown compounds in Table 2) and were tentatively identified as 1-octen-3-ol, 4-ethylphenol, 3-undecen-2-one, decanoic acid, and α-selinene.

### 2.2. In Vivo Study

**Figure 3:**
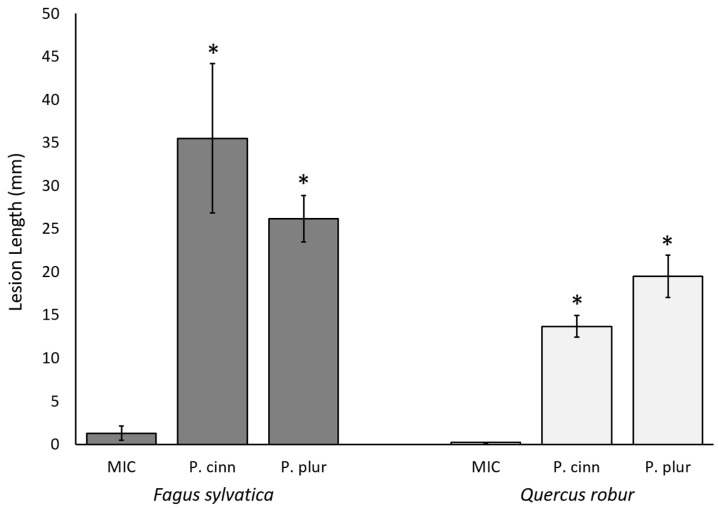
Average stem lesion lengths on *Fagus sylvatica* and *Quercus robur* saplings artificially inoculated with *Phytophthora cinnamomi* (P. cinn) or *Phytophthora plurivora* (P. plur) compared to mock-inoculated controls (MIC). Lesion lengths were recorded 50 days post inoculation. N = 3 for each bar; error bars are ± SD. Asterisks denote significant differences in lesion length compared to the MIC trees of the same species as determined by Dunnett’s test at the α = 0.05 level.

All *Phytophthora*-inoculated trees developed lesions that were significantly larger than those on the MIC trees (Figure 3; see Tables S3 and S4 for statistical analyses). Across both tree species, five compounds in total were detected in the inoculated trees that were not present in the MIC trees (Table 3). Two of these compounds, tentatively identified as anisole and isokaurene, occurred only in the beech trees. Anisole was detected in beech trees infected with either *P. cinnamomi* or *P. plurivora*, but only at 21 dpi. Isokaurene and an unknown compound were detected only in *P. plurivora*-inoculated beech trees at 9 dpi.

In oak trees, an unidentified sesquiterpene was detected at 21 dpi in trees inoculated with either *Phytophthora* species. An unknown compound was also detected at both 9 and 21 dpi, but only in trees inoculated with *P. cinnamomi*.

## 3. Discussion

This work reports diagnostic volatiles from several known *Phytophthora* pathogens of trees in urban and forest landscapes. Many of these pathogens are introduced to new locations via the global trade of live plants, and due to their cryptic nature, are difficult to detect. Discerning VOCs indicative of the presence of *Phytophthora* species may allow for fast and in vivo detection in traded plants. While the in vitro VOC profiles from most of the *Phytophthora* species in our analysis were similar, some species were still easily discernable, and all but *P. cambivora* produced at least one volatile compound that was not present in the other species. Such qualitative differences between species are desirable, because unique compounds could serve as biomarkers of disease and indicate which *Phytophthora* species are present in an unknown sample. These differences would also be useful for chemotaxonomy, particularly for discriminating between closely related species [40,41] and the species complexes that are common in the genus *Phytophthora* [42]. Obtaining a richer blend of in vitro volatiles for biomarker generation could be achieved by using different media with more complex substrates for metabolism [43], something observed by Qiu et al. [44] with *P. cinnamomi*. EMA is a basic medium with only one carbon source and one nitrogen source, not including the amendment β-sitosterol, so there may be a limited capacity for variable VOC production. Future studies comparing species should consider using a blend of nutrients and media constituents, potentially derived from host material to maximize variation.

We hypothesized that in vitro compounds could be useful as biomarkers for detecting infected plants, but none of the in vitro volatiles were observed to differ qualitatively between the infected and mock-inoculated control (MIC) plants. In fact, only five compounds were observed in the *Phytophthora*-infected trees that were not present in the MIC trees. Of these five compounds, two were tentatively identified, anisole and isokaurene; both occurred only in beech. Anisole was detected in beech trees inoculated with *P. cinnamomi* and *P. plurivora*, but other studies examining European beech VOCs have not reported anisole [45,46], including a study looking at VOCs from trees infested with aphids [47]. If anisole is produced only during certain stress events, it may be a useful marker of *Phytophthora* infection in beech trees. Anisole was reported in the roots of hybrid oak (*Quercus petraea* × *Q*. *robur*) after *Melolontha hippocastani* feeding [48], indicating that damage-induced anisole production might be tissue-specific, pest-specific, or both, since it was not seen after stem infection in this study. It is also possible that anisole was produced by the *Phytophthora* species. Anisole is known to be produced by at least one *Penicillium* sp. [49], but its occurrence in *Phytophthora* is unknown. Nonetheless, its occurrence only in the infected beech makes it a potential target for disease diagnosis. Isokaurene has not previously been reported as occurring in European beech either, but it has been induced in maize tissue when infected by different fungi [50]. Isokaurene is a diterpene, and thus, considerably less volatile than most other compounds in this study, so passive sampling methods such as SPME, especially when short sampling times are applied, may not consistently be able to detect it. Isokaurene does, however, have a distinct mass spectrum, meaning it can be unambiguously identified in a sample should it be captured, making it an excellent biomarker in that regard. The other beech-specific volatile was an unknown sesquiterpene, which was only present at the 9 dpi time point. Sesquiterpenes are generally difficult to identify due to their ambiguous mass spectra, and if this compound is only ephemerally present in the early stages of disease, it may not be a suitable biomarker of disease, while if it is consistently present at later time points not examined here, it may still be of value as a biomarker.

In the infected oak trees, two unknowns were detected, an unknown sesquiterpene and an unknown compound with the suggested molecular formula C_14_H_20_O_2_ (Table 3). Of all the in vivo compounds detected for both tree species, the latter unknown compound was the only one present at both sampling time points, and was specific to the *P. cinnamomi*-inoculated trees. The specificity of this unknown to the *P. cinnamomi* treatment and its consistency at both time points make it an ideal candidate for biomarker selection and worthy of further structural elucidation.

In this study, we chose to analyze only compounds that were qualitatively different from the controls in order to increase the likelihood of identifying a viable biomarker that could later be used in targeted and more commercial approaches, such as e-nose devices or ion-mobility spectrometry. In the quest for robust solutions to the burgeoning challenges posed by IASs, and specifically *Phytophthora* spp., finding such “silver bullets” of qualitative differences would present an opportunity for them to be exploited by future VOCs-based tree disease detection methods, marking a new era in plant biosecurity and ecosystem protection. Plants produce and alter their volatile profiles in response to a plethora of different stimuli. Many of these VOCs are shared between different stimuli, are transient, and differ quantitatively depending on the intensity of the stimuli [21,51,52]. Therefore, using compounds that differ quantitatively to differentiate between healthy and diseased plants may lead to erroneous classifications when environmental conditions and other biotic stressors are variable and sampling methods are inconsistent. Brilli et al. [53] successfully used a targeted approach, where plane trees infected with *Ceratocystis platani* were readily distinguishable from healthy controls using a few compounds that were uniquely present in the infected trees. We similarly saw disease-exclusive compounds, but unlike Brilli et al. [53], our unique compounds were likely not from the pathogens themselves. A targeted method may be of limited value in systems with no prominent pathogen-derived VOCs or in pathosystems that do not have any qualitative differences. For example, pine species inoculated with *Fusarium circinatum* could be distinguished from their healthy control plants using SPME-collected VOCs, despite there being no qualitative differences in volatiles between treatments [24]. Whether these quantitative differences are still present under non-laboratory conditions is unknown. Other studies were able to distinguish different disease and insect damage treatments based on differences in volatiles in a variety of tree species [54,55,56,57].

Different tree organs can have different VOC profiles [58,59]. Since some pathogens only attack certain plant organs and substructures with different chemical compositions, volatile profiles associated with damage to a given structure may be sufficiently different for disease diagnosis. Tissue-targeted analyses should increase the sensitivity and specificity of VOCs-based detection methods [60]. Our in vivo sampling method sampled all of the above-ground tissue, but if we had excluded the leaves and only collected VOCs around the inoculation sites, we may have obtained more disease-associated volatiles, and perhaps even some pathogen-derived VOCs. Future studies should consider using a more targeted sampling method that is focused on symptomatic tissues or organs of interest for a certain pathosystem. The extent to which different pests attacking the same tissues can be differentiated is less clear, but some studies have shown that different pests attacking the same tissues emit different VOC profiles [25,26,61,62,63]. In this study, trees infected with *P. cinnamomi* were discernable by VOCs from trees infected with *P. plurivora*, for both oak and beech, even without tissue-targeted sampling. These results further support the contention that pathogens of the same tissue can be differentiated in planta by using volatiles.

Although none of the in vitro *Phytophthora* compounds were found in the in vivo study, some have been reported in other *Phytophthora* pathosystems. For example, 1-octen-3-ol, which was found in *P. polonica* and *P. cambivora* and was an important determinant of *Phytophthora* species from the random forest analysis, was the only compound found at higher levels in the volatiles from solvent extracts of *Phytophthora ramorum*-inoculated *Rhododendron* plants compared to mock-inoculated controls [64]. The C-8 alcohol 1-octen-3-ol is one of the most common fungal volatiles [65]. Its occurrence in oomycetes is less reported, but it was produced by *P. cinnamomi* in culture [66,67].

Hexanal is another common volatile that has been recorded in a number of microbial volatile studies. Interestingly, in Qiu et al. [44], hexanal was observed only in the blank media controls (V8 agar and potato dextrose agar), but not in *P. cinnamomi* colonized media. We observed the opposite, where hexanal was produced by *P. cinnamomi* but was not observed in the control EMA. Hexanal was also detected in *P. cambivora*, *P. gonapodyides*, and *P. polonica* and was identified as an important discriminating compound by PCA. In the in vitro study, this compound was only detected as a minor peak. As hexanal is also prevalent in the environment, its value as a biomarker of disease is limited in practice. Furthermore, our results are in agreement with Qiu et al. [44], in that *Phytophthora cinnamomi* did not produce 4-ethylphenol in culture. However, Qiu et al. [44] did detect 4-ethylphenol from *P. cinnamomi*-infected plants and infested soil, whereas we did not. We did, however, detect 4-ethylphenol from *P. plurivora* cultures in vitro, where it was an important compound for discriminating between species according to the random forest analysis. *Phytophthora plurivora* and *P. cinnamomi* volatiles were also reported by Loulier et al. [66], but none of the compounds they detected for either *P. cinnamomi* or *P. plurivora* were observed by us for the same species. Their methods used a different in vitro growth medium and SPME fiber chemistries, so these differences are not completely surprising, but do demonstrate that volatiles may vary considerably between different setups.

In a study examining the effects of *Phytophthora cactorum* and *P. plurivora* infections on the physiology of hybrid poplar, Ďurkovič et al. [67] found that infected trees emitted germacrene D and α-cubebene from detached leaves, while control trees did not. In this study, neither of these compounds were found solely in the *Phytophthora*-infected trees, but both compounds are known to be emitted by pedunculate oak [68,69] and germacrene D by European beech [70]. Neither germacrene D nor α-cubebene were evident in this study when manually searching for them in the chromatograms of the MIC trees of either species. Since these sesquiterpenes were not found and are known to be emitted by the host trees, they are not considered as suitable biomarkers by our a priori criterion regarding qualitative differences. Furthermore, neither germacrene D nor α-cubebene match either of the unknown C_15_H_24_ compounds in Table 2, despite all being sesquiterpenes (based on tentative molecular formulas and fragmentations), because their respective retention indices are considerably different from those reported for the unknowns with similar mass spectra [71,72].

## 4. Materials and Methods

### 4.1. In Vitro Phytophthora VOC Study

Eight *Phytophthora* species were chosen for the in vitro volatile analysis: *Phytophthora cambivora*, *Phytophthora cinnamomi*, *Phytophthora citricola*, *Phytophthora gonapodyides*, *Phytophthora multivora*, *Phytophthora plurivora*, *Phytophthora polonica*, and *Phytophthora syringae* (see Table S1 for isolate information). All *Phytophthora* isolates were cultivated on Elliott’s medium agar—EMA [73]. Three-millimeter plugs containing hyphae were excised from the margins of actively growing cultures using a sterilized cork borer and transferred to the center of agar slants of EMA amended with β-sitosterol. To make the EMA slants, concentrated β-sitosterol in ethyl acetate (30 mg mL^−1^) was added to cooling but not solidified EMA to reach a final concentration of 10 mg L^−1^ [73], then 5 mL of the amended medium was pipetted into 20 mL glass headspace vials (SU860097, Merck, Darmstadt, Germany). The slants were allowed to cool and solidify before inoculation. The inoculated slants were sealed with headspace vial caps (SU860101, Merck). EMA slants inoculated with sterile EMA plugs were used as non-inoculated controls for identifying background non-Phytophthora-derived volatiles. The vials were incubated at room temperature for 14 or 30 days prior to VOC sampling; no cultures or control vials were sampled at more than one time point. Four replicate vials for each species by time point were used. The sampling time points were chosen based largely on the growth rates of the different *Phytophthora* species and preliminary tests. Most species examined had nearly overgrown the agar slant by day 14, so it was used as an active-growth time point, while day 30 represented a more stagnate-growth metabolism. Volatiles from earlier time points were found to be very similar to those at 14 dpi in preliminary tests, so earlier time points were not used.

Culture volatiles were sampled using divinylbenzene/carboxen/polydimethylsiloxane (DVB/CAR/PDMS) 24 ga SPME fibers (57348-U, Merck) with a 50 μm DVB layer and a 30 μm CAR and PDMS layer. All fibers were conditioned at 260 °C for 5 min before sampling. The fibers were inserted into the vials through the pre-pierced cap septa, and the vials were placed in an incubator maintained at 35 °C and sampled for 24 h. The fibers were manually injected into a 6890 N gas chromatograph (GC) coupled with a 5975 inert mass selective detector (MS, Agilent Technologies, Santa Clara, USA). The injection inlet conditions were splitless, with a temperature of 260 °C and a purge flow of 30 mL min^−1^ for 0.5 min, with an ultra-inert, straight, 2 mm liner. The column was a HP-5 ms ultra inert 60 m, 0.25 mm, 0.25 μm, 7-inch cage (19091S-436UI, Agilent Technologies) with an initial oven temperature of 50 °C. The initial oven temperature of 50 °C was held for 2 min, followed by an 8 °C min^−1^ ramp to 280 °C and a 2.5 min hold. The transfer line temperature was 150 °C. The MS was operated in positive ion mode with a scanning range of 29–500 *m*/*z* and an ion source temperature of 230 °C run at 70 eV. The quadrupole temperature was 150 °C and the detector voltage was 1906 V. An alkane standard mixture C8–C20 (04070, Merck) was also sampled by SPME for 2 h at room temperature in the same headspace vials and injected into the GC-MS using the same parameters for calculating the retention indices.

### 4.2. In Vivo VOC Study

*Phytophthora cinnamomi* and *P. plurivora* were selected for the in vivo inoculation experiments on pedunculate oak (*Quercus robur*) and European beech (*Fagus sylvatica*). The trees were approximately 2 years old, potted in 3 L pots, and maintained in greenhouse conditions with a 16 h light cycle, an average day temperature of 25 °C, and regular watering to runoff for 3 weeks prior to experimentation. The trees were artificially inoculated on either side of the main stem by removing a 1 cm × 0.5 cm piece of bark to expose the xylem. The inoculation points were approximately 5 and 10 cm above the soil line. The excised tissue was replaced by an EMA plug of the same size, taken from the margin of actively growing *Phytophthora* cultures. The mock-inoculated control (MIC) trees were treated with a sterile plug of EMA instead of colonized agar. The inoculation sites were sealed with Parafilm to limit desiccation and contamination. Every treatment and control was run in triplicate for a total of 18 trees.

Volatiles from the inoculated and control trees were analyzed at 9 and 21 days post-inoculation (dpi). These time points were chosen based on lesion development in a pilot inoculation test. Lesions were still small at around 9 dpi, but had grown considerably by 21 dpi. The selected time points attempted to capture an early stage and later stage of symptom development. Parafilm was removed 1 day prior to the day 9 sampling. In preparation for the VOC sampling, a cut Sterilin autoclave bag (11329103, Thermo Fisher Scientific Inc., Waltham, USA) was placed around the stem and soil line to cover the potting mix to limit soil volatiles. The volatiles were collected using the same SPME fibers detailed above, placed in empty, uncapped headspace vials, and secured to the tree near the inoculation point. Immediately after, conditioned fibers were placed in the vials, and the above-ground parts of the trees were encased in another autoclave bag, which was taped shut at the base of the stem and above the cut autoclave bag. The fibers were left for 48 h before being removed and analyzed by GC-MS with the same inlet settings, liner, and column as above. An initial oven temperature of 50 °C was held for 2 min followed by a 5 °C min^−1^ ramp to 200 °C and a 2.5 min hold, followed by a 10 °C min^−1^ ramp to 280 °C with a 2 min hold.

To ensure the inoculations were successful, the bark was gently peeled back using a scalpel to expose the lesions at 50 dpi. The lesion lengths were measured and averaged to obtain a single lesion length per tree.

### 4.3. Data Analysis

For both in vitro and in vivo data sets, GC peaks present in the *Phytophthora*-inoculated treatments but not present in the controls were of greatest interest and further analyzed. This was conducted because we posit that qualitative differences are more relevant than quantitative differences for biomarker selection, particularly given the non-quantitative nature of SPME fibers. To be included, a peak had to be present in at least three of the four replicates for a *Phytophthora* species in the in vitro experiment, and in two of the three treatment replicates in the in vivo experiment, while not being present in any of the MICs. All peak integrations were performed with MSD ChemStation version E.02.02.1431 (Agilent) and the peaks were deconvoluted using AMDIS 32 (NIST). Based on preliminary analyses, a minimum peak area of 11,000 was used for peak calling. The peaks of interest observed in the treatments had their key ions manually searched for in the control specimens to verify their absence. The retention indices for the peaks were calculated based on the retention time of the alkane standards using the calculator from [74]. Tentative identifications were made by matching the mass spectra to compounds in the NIST20 and Wiley12 MS databases and a comparison of known retention indices from verified standards from the literature.

Statistical analyses were performed using RStudio 2023.06.1+524 (Posit). A principal component analysis (PCA) was run on the in vitro compound data sets using the prcomp function to observe the data trends and similarity of the *Phytophthora* VOC profiles. A hierarchical cluster analysis was run with the hclust function on autoscaled data. A random forest analysis was used to determine the in vitro compounds most important for predicting *Phytophthora* species, using the randomForest (with ntree = 500) and caTools packages. A one-way analysis of variance (ANOVA) and a two-tailed Dunnett’s post hoc test were used to determine if the lesion lengths of the *Phytophthora*-inoculated trees differed from those of their respective MICs.

## 5. Conclusions

We demonstrated that *Phytophthora*-infected trees can be distinguished from MIC trees based on the presence of anisole, isokaurene, and a few unidentified VOCs. We also showed that several *Phytophthora* species can be differentiated from each other based on their in vitro volatiles. These compounds have the potential to be used as biomarkers for the development of faster, simpler, and cheaper methods of disease detection, such as e/bio-noses and proton transfer reaction-mass spectroscopy, which offer near real-time analysis. New, higher-throughput methods for identifying diseased plants, particularly those that are still in the asymptomatic phase, are key to limiting the spread of diseased material and safeguarding forests. Volatilomics approaches for disease detection like those used here are one step towards a more secure future in plant health.

## Figures and Tables

**Table 1:** Occurrence of volatiles, denoted by x, after 14 and 30 days of growth for the eight *Phytophthora* species when grown in vitro.

				*P. cambivora*		*P. cinnamomi*		*P. citricola*		*P. gonapodyides*		*P. multivora*		*P. plurivora*		*P. polonica*		*P. syringae*	
Peak No.	MS Library Match	RT ^1^ (min)	KI (KI lit.)	D14	D30	D14	D30	D14	D30	D14	D30	D14	D30	D14	D30	D14	D30	D14	D30
1	2,3-Butanediol	6.19	777 (785) ^1^									x	x						
2	Hexanal	6.53	803 (801) ^2^		x	x	x				x					x	x		
3	2-Furanmethanol	7.48	856 (867) ^1^															x	
4	Unknown 1	9.53	957														x		
5	1-Heptanol	9.83	971 (959) ^2^									x	x						
6	1-Octen-3-ol	10.03	980 (980) ^3^						x							x	x		
7	Unknown 2	10.45	998								x								
8	2,4-Heptadienal ^#^	10.73	1013 (1013) ^2a^		x						x			x	x				
9	Unknown 3	11.23	1039													x			
10	Unknown 4	11.44	1050													x			
11	Unknown 5	11.52	1054	x										x	x				
12	Unknown 6	11.94	1074		x						x								
13	Unknown 7	12.16	1084													x			
14	3,5-Octadien-2-one ^#^	12.4	1095 (1098) ^4^		x						x								
15	2-Nonanol	12.5	1100 (1097) ^2^									x		x	x				
16	3-Nonen-1-ol ^#^	13.57	1157 (1157) ^2b^													x			
17	2,6-Nonadienal ^#^	13.58	1158 (1154) ^2c^		x						x								
18	4-Ethylphenol	13.8	1169 (1178) ^5^											x	x				
19	Unknown 8	13.81	1169													x			
20	1-Nonanol	13.86	1172 (1165) ^2^		x						x		x			x			
21	Unknown 9	14.34	1195		x						x					x			
22	2,4-Nonadienal ^#^	14.74	1218 (1212) ^2a^		x						x					x			
23	Unknown 10	15.06	1236											x	x				
24	4-Decen-1-ol ^#^	15.48	1259 (1259) ^2d^													x			
25	Unknown 11	15.66	1269													x			
26	1-Decanol	15.71	1272 (1266) ^2^	x	x						x	x	x	x	x	x			
27	6-Undecen-2-one ^#^	15.86	1279 (N/A)			x	x		x								x	x	x
28	4-Ethylguaiacol	15.98	1286 (1282) ^6^	x		x	x	x				x		x	x	x	x		
29	2,4-Decadienal (*E*,*Z*)- *	16.17	1296 (1292) ^2^		x						x		x			x	x		
30	2,4-Decadienal (*E*,*E*)- *	16.58	1320 (1319) ^2^		x						x					x	x		
31	3-Undecen-2-one ^#^	16.97	1344 (1344) ^7a^	x	x	x	x			x	x	x	x	x	x	x	x		
32	Methyl 2,4,6-trimethyl benzoate	17.15	1354 (1349) ^8^			x	x												
33	Decanoic acid	17.28	1362 (1366) ^2^	x	x					x	x			x	x				
34	2-Undecenal ^#^	17.36	1367 (1366) ^7b^													x	x		
35	2,4-Undecadien-1-ol ^#^	17.4	1369 (N/A)													x			
36	Unknown 12	17.75	1389		x	x	x			x						x			
37	Unknown 13	18.17	1414							x	x								
38	Unknown 14	18.37	1428							x									
39	1-Phenyl-2-hexanone	18.7	1449 (N/A)									x	x						
40	2,6-Dodecadienal ^#^	18.77	1454 (1445) ^2c^								x								
41	Unknown 15	19.25	1483					x	x									x	
42	Unknown 16	19.29	1486	x						x		x	x						
43	2-Tridecanol	19.56	1503 (1510) ^9^										x						
44	Aristolochene	19.62	1507 (1487) ^10^			x	x												
45	Unknown 17	19.84	1522		x						x								
46	Unknown 18	20.24	1549					x											
47	Dodecanoic acid	20.43	1562 (1565) ^2^		x						x		x						
48	1-Tridecanol	20.67	1578 (1570) ^2^										x						
49	Unknown 19	20.82	1587													x			
50	Unknown 20	20.92	1594	x								x	x						
51	Unknown 21	21.33	1622														x		
52	1-Tetradecanol	22.17	1681 (1671) ^2^	x								x	x						
53	6-Pentadecen-2-one ^#^	22.21	1684 (1667) ^2b^										x						
54	γ-Dodecalactone	22.31	1691 (1676) ^2^			x				x	x		x			x	x		
55	δ-Dodecalactone	22.76	1724 (1704) ^2^	x						x	x								
56	1-Hexadecanol	24.86	1845 (1874) ^2^										x						
57	Unknown 22	25.55	1874								x								
58	Unknown 23	25.62	1876								x								

RT = retention time; KI = Kovâts index; lit. = KI from literature that used authentic standards for KI calculations. D14 = 14 days post-inoculation; D30 = 30 days post-inoculation. x = present in the species at that given time point, absent in all controls. * = suggested isomer based on KI values and MS match. ^#^ = cis-trans isomerism cannot be confirmed. ^1^ Ames et al. [29]; ^2^ Adams [30] [a = (2*E*, 4*E*)-isomer; b = (*Z*)-isomer; c = (2*E*, 6*Z*)-isomer]; d = (*E*)-isomer KI 1259 or (*Z*)-isomer KI 1262; ^3^ Beaulieu et al. [31]; ^4^ Lozano et al. [32]; ^5^ Martí et al. [33]; ^6^ Steinhaus and Schieberle [34]; ^7^ Lazarević et al. [35] [a =isomer unspecified; b = (*2E*)-isomer]; ^8^ Rostad and Pereira [36]; ^9^ Ohnishi and Shibamoto [37]; ^10^ Retta et al. [38].

**Table 2:** The five most important compounds for distinguishing *Phytophthora* species from each other based on principal components (PC) 1 and 2 and the random forest (RF) analysis in the full set of in vitro produced compounds and in which species they were detected.

Peak No.	Tentative ID	Selection Criteria	Species Detected in
2	Hexanal	PC1	camb, cinn, gona, polo
6	1-Octen-3-ol	RF	citr, polo
10	Unknown 4	PC2	polo
13	Unknown 7	PC2	polo
16	3-Nonen-1-ol	PC2	polo
17	2,6-Nonadienal	PC1	camb, gona
18	4-Ethylphenol	RF	plur
22	2,4-Nonadienal	PC1	camb, gona, polo
25	Unknown 11	PC2	polo
31	3-Undecen-2-one	PC1, RF	camb, cinn, gona, mult, plur, polo
33	Decanoic acid	RF	camb, gona, plur
34	2-Undecenal	PC2	polo
44	Aristolochene	RF	cinn
45	Unknown 17	PC1	camb, gona

*Phytophthora* species abbreviations: camb = *P. cambivora*; cinn = *P. cinnamomi*; gona = *P. gonapodyides*; mult = *P. multivora*; plur = *P. plurivora*; polo = *P. polonica*.

**Table 3:** Tentatively identified in vivo compounds uniquely present in *Phytophthora*-inoculated trees.

Tree	Pathogen	Compound	MF	RT (min)	KI (KI lit.)	9 dpi	21 dpi
*Quercus robur*	*P. cinnamomi*	Unknown 1	C_14_H_20_O_2_	25.10	1445	x	x
		Unknown 3	C_15_H_24_	30.25	1661		x
	*P. plurivora*	Unknown 3	C_15_H_24_	30.25	1661		x
*Fagus sylvatica*	*P. cinnamomi*	Anisole	C_7_H_8_O	10.00	922 (913) ^1^		x
	*P. plurivora*	Anisole	C_7_H_8_O	10.00	922 (913) ^1^		x
		Unknown 2	C_15_H_24_	29.48	1627	x	
		Isokaurene	C_20_H_32_	38.21	1964 (1988) ^2^	x	

MF = suggested molecular formula; RT = retention time; dpi = days post-infection; KI = Kovâts index; lit. = KI from literature that used authentic standards for KI determinations. x = present in inoculated trees, not detected in controls. ^1^ Adams [30]; ^2^ Skaltsa et al. [39].

## Data Availability

The data that support the findings of this study are available on request from the corresponding author.

## Supplementary Materials

The following supporting information can be downloaded at: https://www.mdpi.com/article/10.3390/molecules29081749/s1, Figure S1: Mass spectra of important unidentified compounds 4, 7, 11, and 17 in Table 2 from the in vitro study; Figure S2: Mass spectra of unidentified compounds 1–3 in Table 3 from the in vivo study; Table S1: Phytophthora species used in the study; Table S2: Random forest output; Table S3: R outputs of ANOVA tables and Dunnett’s test for lesion lengths in pedunculate oak trees; Table S4: R outputs of ANOVA tables and Dunnett’s test for lesion lengths in European beech trees.
