# Supplementary material for: Detecting Pathogenic Phytophthora Species Using Volatile Organic Compounds
Source: Molecules. 2024 Apr 12;29(8):1749. doi: 10.3390/molecules29081749 (PMC11052055; doi:10.3390/molecules29081749)
Supplement: Supplementary file 1 [file molecules-29-01749-s001.zip › molecules-2849836-supplementary.pdf]

Supplemental Material for "Detecting pathogenic *Phytophthora* species using volatile organic compounds"

Authors: Patrick Sherwood[1], Ida Nordström[1], Stephen Woodward[2], Björn Bohman[3], and Michelle Cleary[1]

Affiliations:

[1]Southern Swedish Forest Research Centre, Swedish University of Agricultural Sciences, Box 190, 234 22 Lomma, Sweden
[2]Department of Plant Protection Biology, Swedish University of Agricultural Sciences, Box 102, 234 22 Lomma, Sweden
[3]School of Biological Sciences, Department of Plant and Soil Science, University of Aberdeen, Cruickshank Building, St. Machar Drive, Aberdeen AB24 3UU, UK

Table S1. *Phytophthora* species used in the study.

Species	Isolate	Genbank Accession
P. cambivora	A02	PP082873
P. cinnamomi	A04	MT663321
P. citricola	A23	MT663314
P. gonapodyides	P27#100	KX055998
P. multivora	A21	PP082874
P. plurivora	A03	MT663323
P. polonica	UKR_Ph_10	MT420386.1
P. syringae	P26 L53	PP082875

Table S2. Random forest output

```
Call:
 randomForest(x = complete_train[-59], y = complete_train$Species,      ntree
= 500)
               Type of random forest: classification
                     Number of trees: 500
No. of variables tried at each split: 7

        OOB estimate of error rate: 6.82%
Confusion matrix:
             cambivora cinnamomi citricola gonapodyides multivora plurivora
cambivora            5         0         0            0         0         0
cinnamomi            0         7         0            0         0         0
citricola            0         0         3            0         0         0
gonapodyides         0         0         0            7         0         0
multivora            0         0         0            0         3         0
plurivora            0         0         0            0         0         6
polonica             0         0         0            0         0         0
syringae             0         0         0            0         0         0
             polonica syringae class.error
cambivora           0        0         0.0
cinnamomi           0        0         0.0
citricola           0        2         0.4
gonapodyides        0        0         0.0
multivora           0        0         0.0
plurivora           0        0         0.0
polonica            4        1         0.2
syringae            0        6         0.0



y_pred
               cambivora cinnamomi citricola gonapodyides multivora plurivora
  cambivora            3         0         0            0         0         0
  cinnamomi            0         1         0            0         0         0
  citricola            0         0         3            0         0         0
  gonapodyides         0         0         0            1         0         0
  multivora            0         0         0            0         5         0
  plurivora            0         0         0            0         0         2
  polonica             0         0         0            0         0         0
  syringae             0         0         0            0         0         0
              y_pred
               polonica syringae
  cambivora           0        0
  cinnamomi           0        0
  citricola           0        0
  gonapodyides        0        0
  multivora           0        0
  plurivora           0        0
  polonica            3        0
  syringae            0        2


Confusion Matrix and Statistics

              Reference
Prediction     cambivora cinnamomi citricola gonapodyides multivora plurivora
  cambivora            3         0         0            0         0         0
  cinnamomi            0         1         0            0         0         0
  citricola            0         0         3            0         0         0
  gonapodyides         0         0         0            1         0         0
```

```
  multivora            0          0          0             0          5          0
  plurivora            0          0          0             0          0          2
  polonica             0          0          0             0          0          0
  syringae             0          0          0             0          0          0
              Reference
Prediction     polonica syringae
  cambivora           0        0
  cinnamomi           0        0
  citricola           0        0
  gonapodyides        0        0
  multivora           0        0
  plurivora           0        0
  polonica            3        0
  syringae            0        2

Overall Statistics

               Accuracy : 1
                 95% CI : (0.8316, 1)
    No Information Rate : 0.25
    P-Value [Acc > NIR] : 9.095e-13

                  Kappa : 1

 Mcnemar's Test P-Value : NA

Statistics by Class:

                     Class: cambivora Class: cinnamomi Class: citricola
Sensitivity                      1.00             1.00             1.00
Specificity                      1.00             1.00             1.00
Pos Pred Value                   1.00             1.00             1.00
Neg Pred Value                   1.00             1.00             1.00
Prevalence                       0.15             0.05             0.15
Detection Rate                   0.15             0.05             0.15
Detection Prevalence             0.15             0.05             0.15
Balanced Accuracy                1.00             1.00             1.00
                     Class: gonapodyides Class: multivora Class: plurivora
Sensitivity                         1.00             1.00              1.0
Specificity                         1.00             1.00              1.0
Pos Pred Value                      1.00             1.00              1.0
Neg Pred Value                      1.00             1.00              1.0
Prevalence                          0.05             0.25              0.1
Detection Rate                      0.05             0.25              0.1
Detection Prevalence                0.05             0.25              0.1
Balanced Accuracy                   1.00             1.00              1.0
                     Class: polonica Class: syringae
Sensitivity                     1.00             1.0
Specificity                     1.00             1.0
Pos Pred Value                  1.00             1.0
Neg Pred Value                  1.00             1.0
Prevalence                      0.15             0.1
Detection Rate                  0.15             0.1
Detection Prevalence            0.15             0.1
Balanced Accuracy               1.00             1.0
```

Compound Number	MeanDecreaseGini
31	2.21601429
33	2.08287808
44	2.00814904
28	1.64924756
18	1.35843650

37	1.34925174
32	1.22850601
23	1.21609388
6	1.19249060
54	1.19190446
55	1.19120649
27	1.14697671
34	1.13825623
15	1.13415435
2	1.11860148
41	1.11393595
26	1.04633624
11	0.89502606
42	0.74019379
36	0.71852546
29	0.70871442
30	0.66924751
5	0.57513530
46	0.55359017
8	0.53217223
50	0.48836867
52	0.45715472
1	0.39709090
39	0.38468962
3	0.32260217
20	0.30363248
47	0.24822879
12	0.24086448
57	0.23562026
7	0.23367772
17	0.22658870
38	0.22108477
51	0.20001909
22	0.18427660
14	0.18345722
45	0.16987478
40	0.16830214
21	0.14493169
58	0.13438665
4	0.12300568
16	0.05468539
24	0.04792784
48	0.03992667
49	0.03786164
9	0.03621675
43	0.03426730
19	0.03391870
56	0.03299841
53	0.02790879
10	0.02785695
13	0.02250193
35	0.01694943
25	0.00000000

Compound numbers correspond to the peak number column in Table 1 of the main text.

R outputs of ANOVA tables and Dunnett's test for lesion lengths by tree species. Note that Inocul = inoculation type, which has 3 levels (1) MIC, (2) cinnamomi and (3) plurivora.

Table S3 R outputs of ANOVA tables and Dunnett's test for lesion lengths in pedunculate oak trees

```
> #oak
> model_oak <- aov(Lesion ~ Inocul, data = data_oak)
summary(model_oak)
            Df Sum Sq Mean Sq F value  Pr(>F)    
Inocul       2  601.1  300.53   79.55 4.8e-05 ***
Residuals    6   22.7    3.78                    
---
Signif. codes:  0 '***' 0.001 '**' 0.01 '*' 0.05 '.' 0.1 ' ' 1

> DunnettTest(x=data_oak$Lesion, g=data_oak$Inocul)

  Dunnett's test for comparing several treatments with a control :  
    95% family-wise confidence level

$aMIC
                   diff    lwr.ci   upr.ci    pval    
cinnamomi-MIC 13.66667  9.118414 18.21492 0.00024 ***
plurivora-MIC 19.50000 14.951747 24.04825 3.2e-05 ***

---
Signif. codes:  0 '***' 0.001 '**' 0.01 '*' 0.05 '.' 0.1 ' ' 1
```

Table S4 R outputs of ANOVA tables and Dunnett's test for lesion lengths in European beech trees

```
> #beech
> model_beech <- aov(Lesion ~ Inocul, data = data_oak)
> summary(model_beech)
            Df Sum Sq Mean Sq F value  Pr(>F)    
Inocul       2  601.1  300.53   79.55 4.8e-05 ***
Residuals    6   22.7    3.78                    
---
Signif. codes:  0 ‘***’ 0.001 ‘**’ 0.01 ‘*’ 0.05 ‘.’ 0.1 ‘ ’ 1
> DunnettTest(x=data_beech$Lesion, g=data_beech$Inocul)

  Dunnett's test for comparing several treatments with a control :  
    95% family-wise confidence level

$aMIC
                   diff    lwr.ci   upr.ci   pval    
cinnamomi-MIC 33.70000 18.459461 48.94054 0.0013 ** 
plurivora-MIC 24.36667  9.126128 39.60721 0.0067 ** 

---
Signif. codes:  0 '***' 0.001 '**' 0.01 '*' 0.05 '.' 0.1 ' ' 1
```

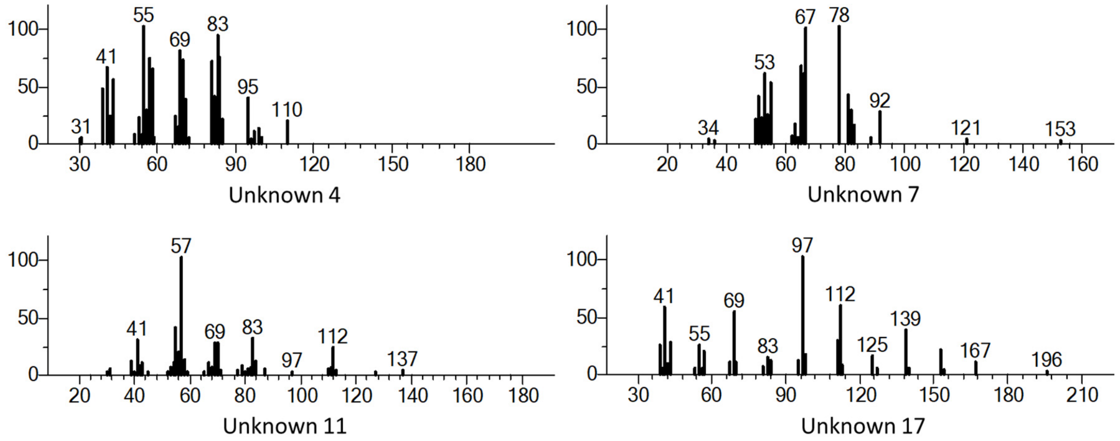

Figure S1. Mass spectra of important unidentified compounds 4, 7, 11, and 17 in Table 2 from the *in vitro* study.

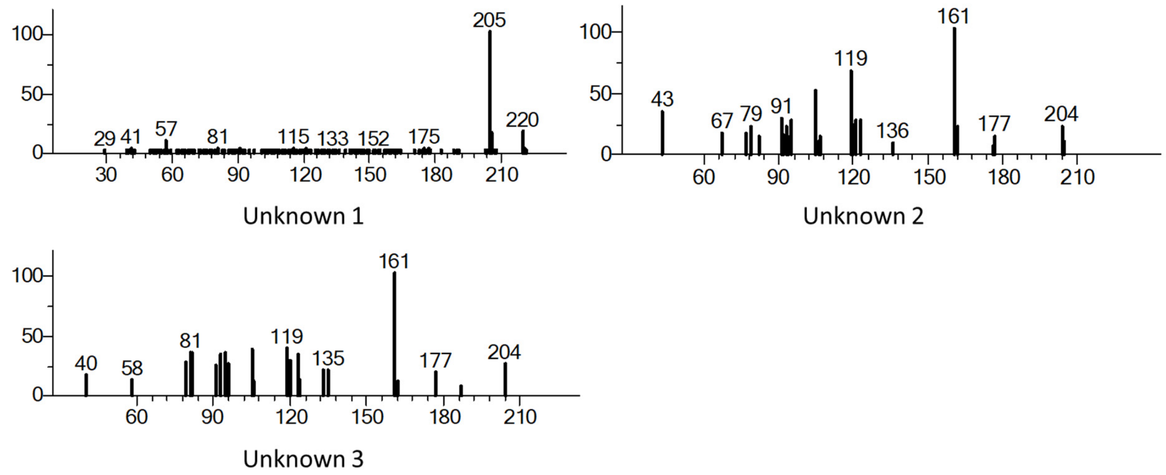

Figure S2. Mass spectra of unidentified compounds 1-3 in Table 3 from the *in vivo* study
